# Supplementary material for: Hydrogenation of different carbon substrates into light hydrocarbons by ball milling
Source: Nat Commun. 2023 Aug 29;14:5257. doi: 10.1038/s41467-023-40915-5 (PMC10465506; doi:10.1038/s41467-023-40915-5)
Supplement: Supplementary file 1 — Supplementary Information [file 41467_2023_40915_MOESM1_ESM.pdf]

Supplementary Information

Hydrogenation of different carbon substrates into light hydrocarbons by ball milling

Linfeng Li[1,3], Olena Vozniuk[1,3]*, Zhengwen Cao[1,2], Pit Losch[1], Michael Felderhoff[1] and Ferdi Schüth[1*]

[1]Department of Heterogeneous Catalysis, Max-Planck-Institut für Kohlenforschung, Kaiser-Wilhelm-Platz 1, 45470 Mülheim an der Ruhr, Germany.[2]Qingdao Institute of Bioenergy and Bioprocess Technology, Chinese Academy of Sciences, Qingdao Key Laboratory of Functional Membrane Material and Membrane Technology, No.189 Songling Road, 266101 Qingdao, China.[3]These authors contributed equally: Linfeng Li, Olena Vozniuk. *e-mail: schueth@kofo.mpg.de olena.vozniuk@dupont.com

This Supplementary Information includes:

Supplementary figures 1 - 6 (Page 1 - 6)

Supplementary tables 1 - 4 (Page 7 - 10)

Supplementary data of High-Resolution Mass Spectrometry Entry 1 - 6 (Page 11- 26)

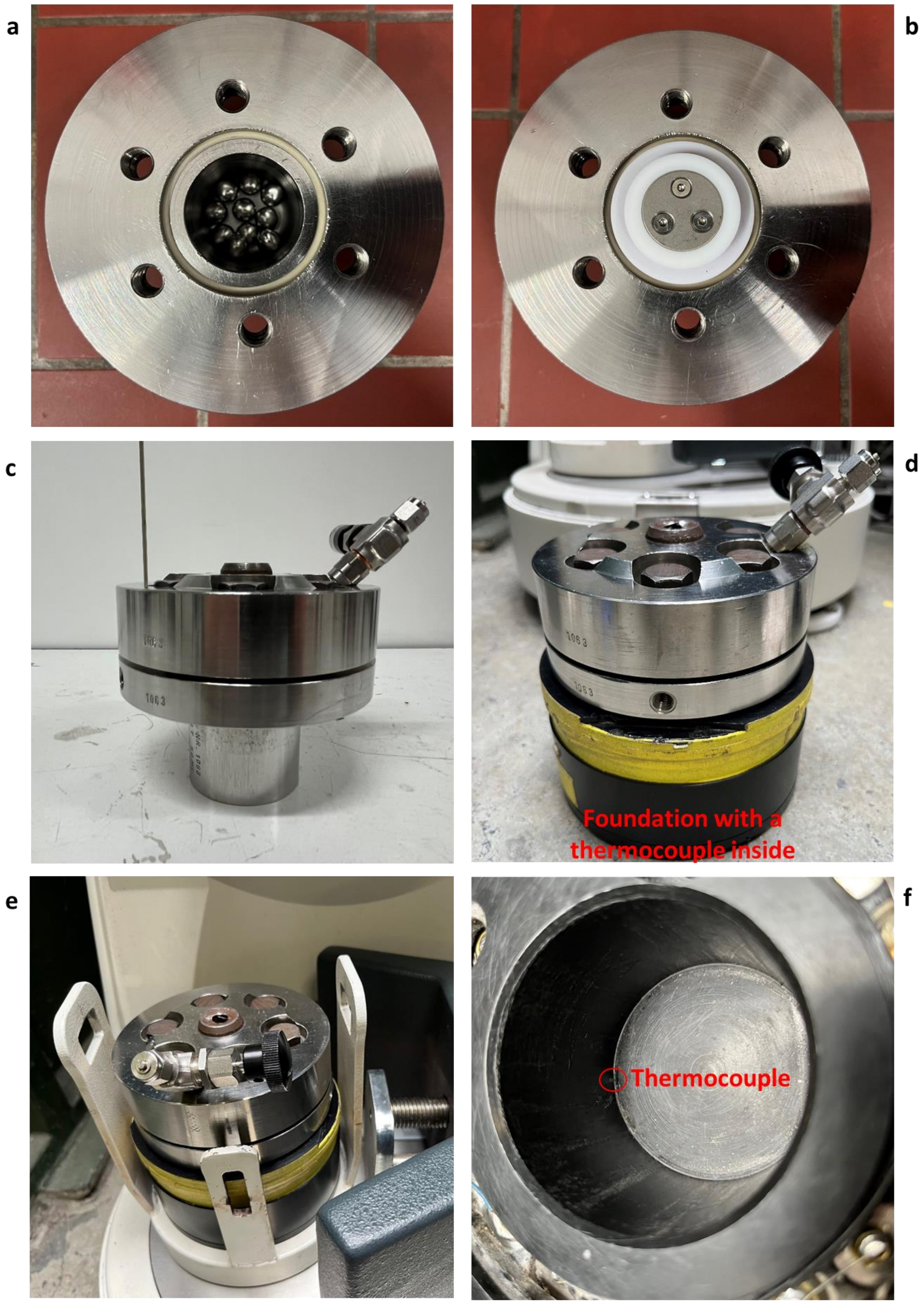

Fig. S1 | Images of the milling jar for ball milling hydrogenation. **a,** Top-view image of the milling jar loaded with 10 10 mm stainless steel balls. **b,** Top-view image of the milling jar capped by Teflon-lid. **c,** Image of milling jar completely sealed. **d,** Image of milling jar embedded in the foundation. **e**, Image of milling jar embedded in foundation on the planetary mill. **f,** Image of the thermocouple in the foundation.

a **b**

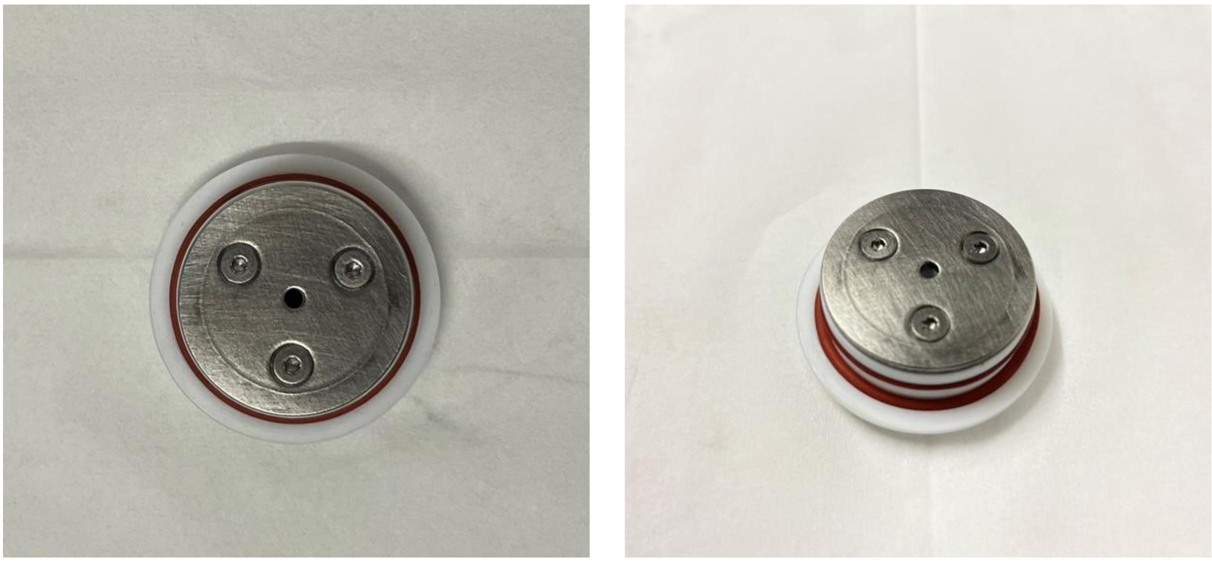

Fig. S2 | Images of the Teflon-lid. a, Top-view image of Teflon-lid. **b,** Side-view Teflon-lid. The red ring is for sealing.

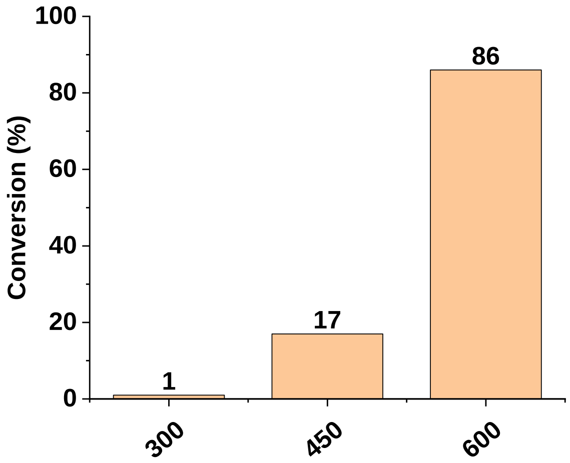

Fig. S3 | The conversion for different rotational speeds. Reaction condition: 300 rpm, 450 rpm and 600 rpm, 7 hours of milling time, 170 bar of H_2, 10 10 mm stainless steel balls, 50 mg of AC, 200 mg of Fe, 300 mg of γ-Al_2O_3.

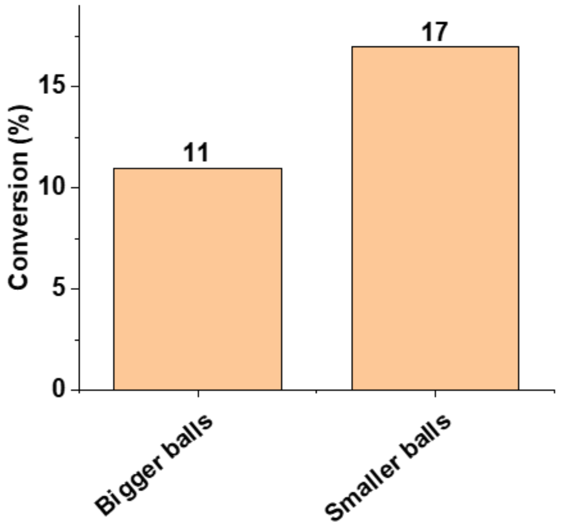

Fig. S4 | The conversion for different sizes of balls. Reaction condition: Bigger balls**:** 3 10 mm balls, 2 12 mm balls and 1 15 mm ball. Smaller balls: 10 10 mm balls. All the balls are stainless steel. 450 rpm, 7 hours of milling time, 170 bar of H_2, 50 mg of AC, 200 mg of Fe, 300 mg of γ-Al_2O_3.

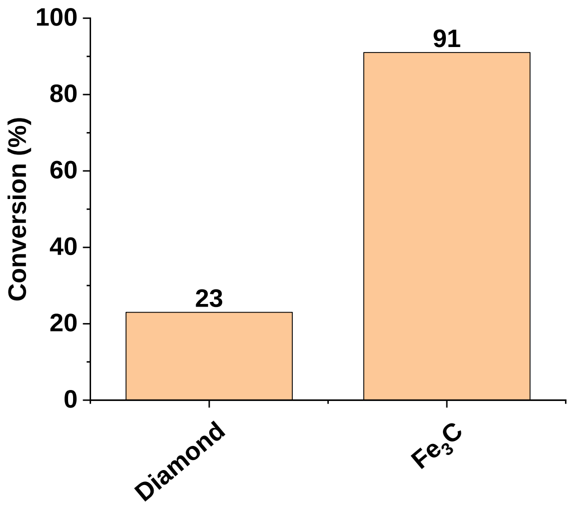

Fig. S5 | The conversion of diamond and Fe_3C. Reaction condition for diamond: 450 rpm, 21 hours of milling time, 170 bar of H_2, 10 10 mm stainless steel balls, 200 mg of Fe, 300 mg of γ-Al2O3, 50 mg of diamond. Reaction condition for Fe_3C: 450 rpm, 7 hours of milling time, 170 bar of H_2, 10 10 mm stainless steel balls, 215 mg of Fe_3C, 300 mg of γ-Al_2O_3.

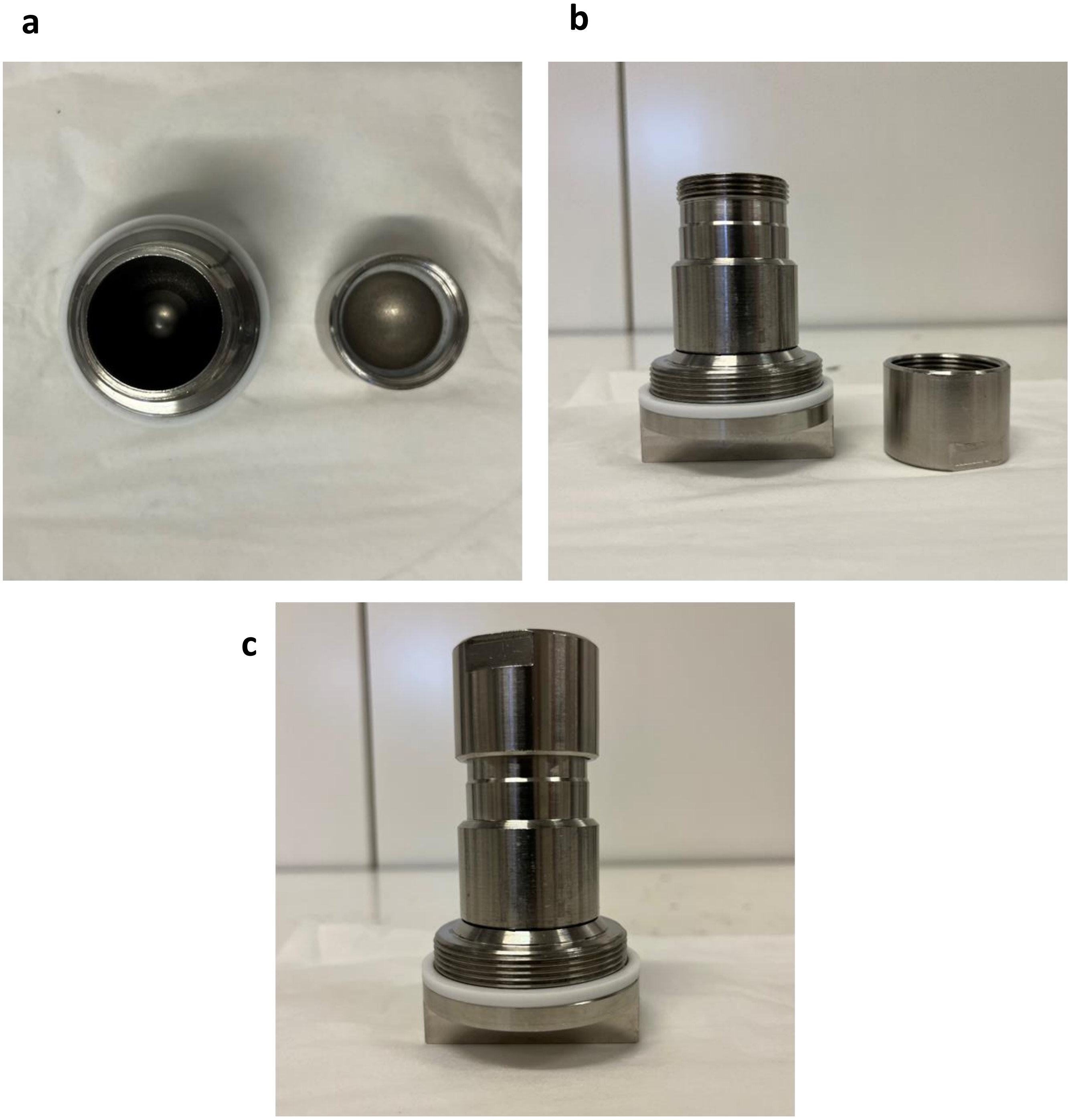

Fig. S6 | Images of the milling jar for freezing milling. a, Top-view image of the milling jar loaded with 1 15 mm stainless steel ball. **b,** Side-view image of the milling jar. **c,** Image of milling jar completely sealed.

Table S1 | The results of element analysis. The carbon content was used for calculating conversion.

Carbon substrates	C, %w	H, %w	O, %w
Polyethylene (PE)	85.61	14.31	
Polyethylene terephthalate (PET)	62.42	4.15	33.35
Spruce wood	48.49	6.58	33.89
Anthracite	94.36	1.83	2.74
Hard coal	82.17	5.23	4.97
Active carbon (AC)	91.95	3.10	2.87

Table S2 | The selectivity to C_1-C_4 alkane for 7-hour milling. Reaction conditions: 450 rpm, 170 bar of H_2, 10 10 mm stainless steel balls, 50 mg of carbon substrates, 200 mg of Fe, 300 mg of γ-Al_2O_3.

Selectivity (%)	C1	C2	C3	C4
PE	93.41	4.45	1.31	0.83
PET	80.28	14.96	3.02	1.74
Spruce wood	79.93	9.97	6.54	3.57
Anthracite	94.84	3.98	0.95	0.24
Hard coal	91.58	5.51	1.96	0.96
Active carbon	92.18	5.42	1.80	0.60

Table S3 | The selectivity to C_1-C_4 alkane for 21-hour milling. Reaction condition: 450 rpm, 170 bar of H_2, 10 10 mm stainless steel balls, 50 mg of carbon substrates, 200 mg of Fe, 300 mg of γ-Al_2O_3.

Selectivity (%)	C1	C2	C3	C4
PE	94.52	3.36	1.42	0.71
PET	97.16	2.84	0.00	0.00
Spruce wood	96.01	3.66	0.33	0.00
Anthracite	98.23	1.77	0.00	0.00
Hard coal	97.80	2.20	0.00	0.00
Active carbon	98.20	1.80	0.00	0.00

Table S4 | TEMPO trapping experiments. D represents detected. ND represents not detected. N.A. represents the peaks intensity are very low and overlaps with peaks of other ions, and a clear pattern is not available, therefore the molecule is unable to be identified. Reaction condition: 450 rpm, 7 hours of milling time, 10 10 mm stainless steel balls, 25 mg of TEMPO. **Entry 1**. 200 mg of Fe, 300 mg of γ-Al_2O_3, 50 mg of AC, 170 bar of H_2. **Entry 2.** 200 mg of Fe, 300 mg of γ-Al_2O_3, 50 mg of PE, 170 bar of H_2. **Entry 3.** 200 mg of Fe, 300 mg of γ-Al_2O_3, 50 mg of PE, 50 bar of Ar. **Entry 4.** 300 mg of γ-Al_2O_3, 50 mg of PE, 50 bar of Ar. **Entry 5.** 200 mg of Fe, 50 mg of PE, 50 bar of Ar. **Entry 6.** 200 mg of Cu, 300 mg of γ-Al_2O_3, 50 mg of PE, 50 bar of Ar. The original HR-MS results for every entry are in supplementary data of HR-MS. m/z (C_1-TEMPO) = 172, m/z (C_2-TEMPO) = 186, m/z (C_3-TEMPO) = 200, m/z (C_4-TEMPO) = 214, m/z (C_5-TEMPO) = 228, m/z (C_6-TEMPO) = 242.

Entry	C_1-TEMPO	C_2-TEMPO	C_3-TEMPO	C_4-TEMPO	C_5-TEMPO	C_6-TEMPO
1	D	D	D	D	D	ND
2	D	D	D	D	D	D
3	D	D	D	D	D	D
4	ND	ND	ND	ND	ND	ND
5	D	D	D	D	D	D
6	N.A.	ND	ND	ND	ND	ND

Entry 1

Elektrospray-ionisation pos. ions

Characteristicial ions:
142 = [C9H20N1]+

Additionally characteristical ions:
359

11.01.2022
File: E34885b-00.RAW
Analyse: LIQ-LA-056-09
STF: Li, Linfeng

Messung:	API-MS
Ionisierung:	ESIpos
Lösungsmittel:	CH2Cl2+CH3CN+CH3COOH
Spektrometer:	Q Exactive Plus Orbitrap
ELNA:	
Auswerter:	Kohler (2243)

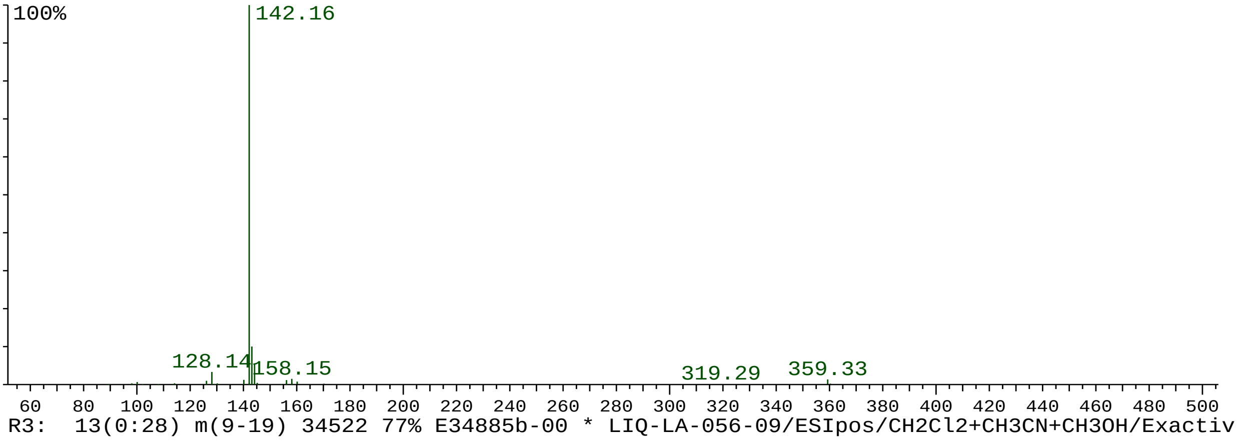

R3: 13(0:28) m(9-19) 34522 77% E34885b-00 * LIQ-LA-056-09/ESIpos/CH2Cl2+CH3CN+CH3OH/Exactiv

reduced mass range m/z 145 - 340

Characteristicial ions:
156 = [C9H18N1O1]+
158 = [C9H20N1O1]+ = [157 + H]+
172 = [C10H22N1O1]+ = [171 + H]+
186 = [C11H24N1O1]+ = [185 + H]+
200 = [C12H26N1O1]+ = [199 + H]+
214 = [C13H28N1O1]+ = [213 + H]+
228 = [C14H30N1O1]+ = [227 + H]+

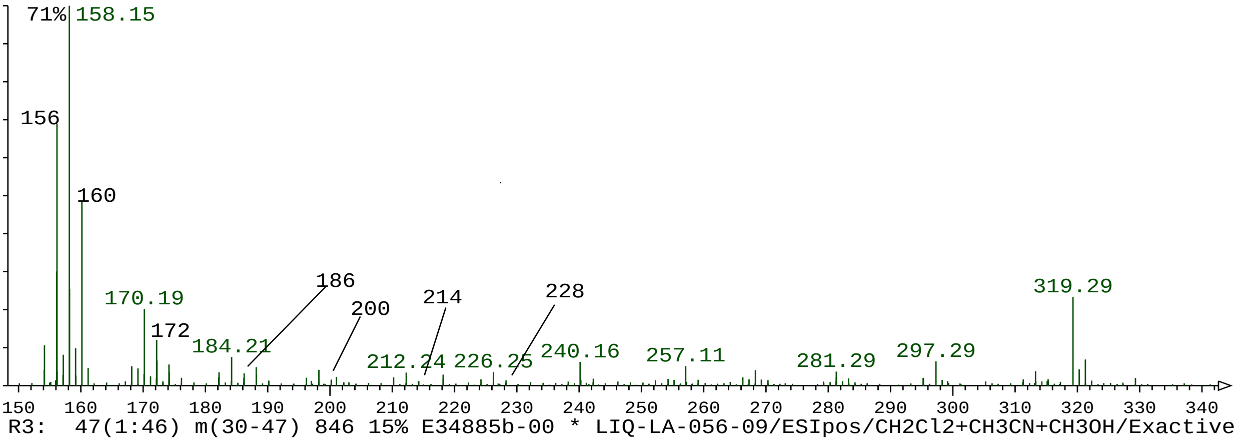

R3: 47(1:46) m(30-47) 846 15% E34885b-00 * LIQ-LA-056-09/ESIpos/CH2Cl2+CH3CN+CH3OH/Exactive

 *** Angegebene Mol.-Gewichte u. Massenzahlen basieren auf dem häufigsten Isotop der Elemente ***

Entry 1

Mass to be matched (m/z): 158.153890 Charge: 1

Mass Tolerance: ±0.005000
Restriction of atom numbers:

C	H	N	O
1-110	1-100	1-3	1-5

Number of calculated Formulas: 2

Formula	Diff.(ppm)	theor. m/z
C9 H20 N1 O1	0.31	158.153939
C4 H20 N3 O3	-25.13	158.149916

11.01.2022
File: E34885b-00.RAW
Analyse: LIQ-LA-056-09
STF: Li, Linfeng

Messung: API-MS (HRMS)
Ionisierung: ESIpos
Lösungsmittel: CH2Cl2+CH3CN+CHC3OOH
Spektrometer: Q Exactive Plus Orbitrap
ELNA:

Auswerter: Kohler (2243)

Suggestion:
C9H19N1O1 MW 157

Characteristicial ions:
158 = [157 + H]+

Mass to be matched (m/z): 172.169420 Charge: 1

Mass Tolerance: ±0.005000
Restriction of atom numbers:

C	H	N	O
1-110	1-100	1-3	1-5

Number of calculated Formulas: 2

Formula	Diff.(ppm)	theor. m/z
C10 H22 N1 O1	0.98	172.169589
C5 H22 N3 O3	-22.38	172.165566

Suggestion:
C10H21N1O1 MW 171

Characteristicial ions:
172 = [171 + H]+

Mass to be matched (m/z): 186.185090 Charge: 1

Mass Tolerance: ±0.005000
Restriction of atom numbers:

C	H	N	O
1-110	1-100	1-3	1-5

Number of calculated Formulas: 2

Formula	Diff.(ppm)	theor. m/z
C11 H24 N1 O1	0.80	186.185239
C6 H24 N3 O3	-20.81	186.181216

Suggestion:
C11H23N1O1 MW 185

Characteristicial ions:
186 = [185 + H]+

Mass to be matched (m/z): 200.200710 Charge: 1

Mass Tolerance: ±0.005000
Restriction of atom numbers:

C	H	N	O
1-110	1-100	1-3	1-5

Number of calculated Formulas: 2

Formula	Diff.(ppm)	theor. m/z
C12 H26 N1 O1	0.89	200.200889
C7 H26 N3 O3	-19.20	200.196866

Suggestion:
C12H25N1O1 MW 199

Characteristicial ions:
200 = [199 + H]+

Entry 1

```
Mass to be matched (m/z): 214.216290  Charge: 1

Mass Tolerance: ±0.005000
Restriction of atom numbers:
C     H     N     O
1-110 1-100 1-3   1-5
Number of calculated Formulas:   2

Formula           Diff.(ppm)    theor. m/z
C13 H28 N1 O1           1.16    214.216539
C8 H28 N3 O3          -17.62    214.212516

              Suggestion:
              C13H27N1O1  MW 213

              Characteristicial ions:
              214 = [213 + H]+
```

```
Mass to be matched (m/z): 228.231950  Charge: 1

Mass Tolerance: ±0.005000
Restriction of atom numbers:
C     H     N     O
1-110 1-100 1-3   1-5
Number of calculated Formulas:   2

Formula           Diff.(ppm)    theor. m/z
C14 H30 N1 O1           1.05    228.232189
C9 H30 N3 O3          -16.58    228.228166

            Suggestion:
            C14H29N1O1   MW 227

            Characteristicial ions:
            228 = [227 + H]+
```

Entry 2

```
electrospray-ionization (Sol.: CH2Cl2+CH3CN+CH3COOH )
pos. ions

characteristical ions
142 = [C9H20N1]+
```

Datum	17.11.2021
File:	156448d-00.RAW
ELNA:	34229
Analyse:	LIQ-LA-064-01
STF: Li, Linfeng	
Ionisierung:	ESIpos
Lösungsmittel:	CH2Cl2+CH3CN+CH3COOH
Spektrometer:	Exactive
Auswerter:	Kampen (2242)

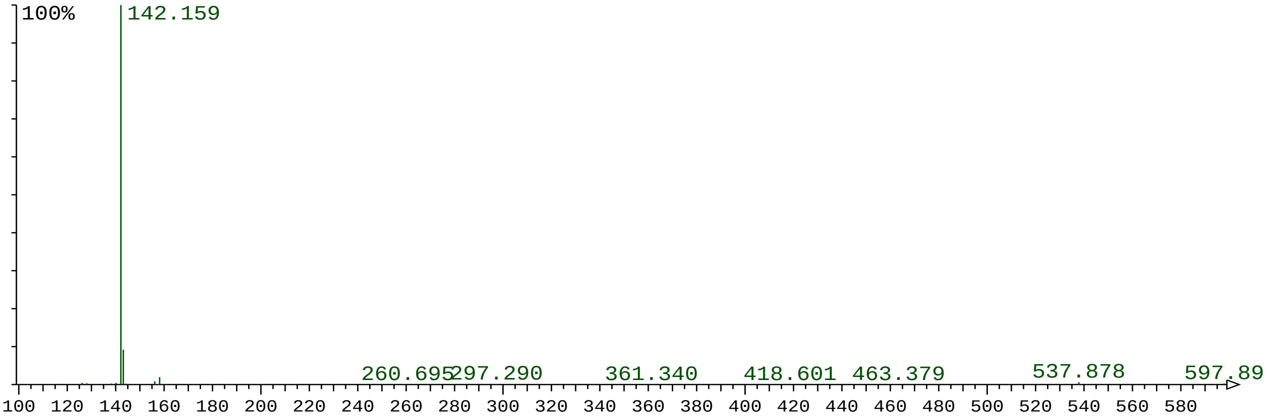

133(8:51) 96176 82% 156448d-00 * LIQ-LA-064-01/ESIpos/CH2Cl2+CH3CN+CH3COOH/Exactive/34229/

```
reduced mass range m/z 150-250

characteristical ions
156 = [C9H18N1O1]+
158 = [C9H20N1O1]+ = [157 + H]+
172 = [C10H21N1O1]+ = [171 + H]+
186 = [C11H24N1O1]+ = [185 + H]+ (low intensity)
200 = [C12H26N1O1]+ = [199 + H]+
214 = [C13H28N1O1]+
228 = [C14H30N1O1]+
242 = [C15H32N1O1]+
```

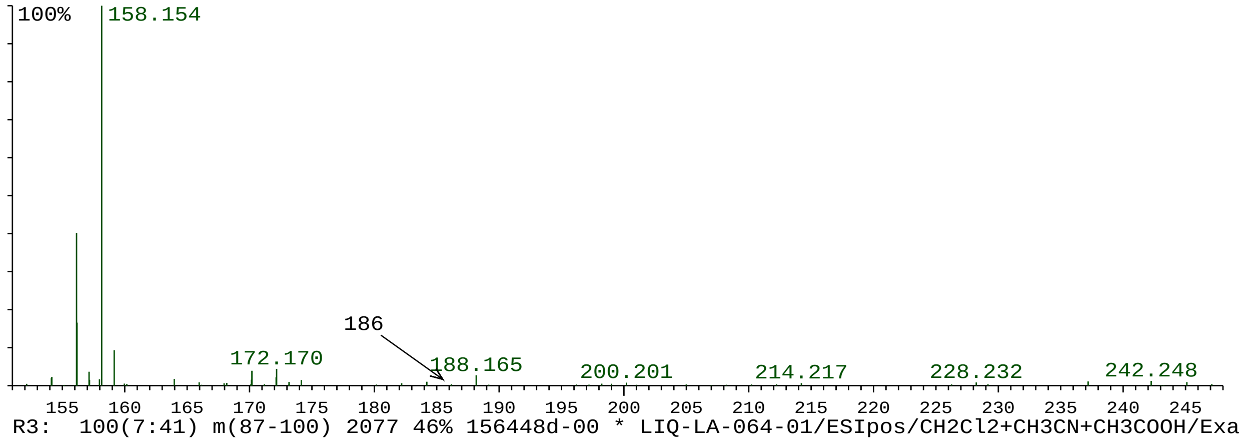

R3: 100(7:41) m(87-100) 2077 46% 156448d-00 * LIQ-LA-064-01/ESIpos/CH2Cl2+CH3CN+CH3COOH/Exa

Entry 2

```
17.11.2021
File: 156448d-00.RAW
Analyse: LIQ-LA-064-01
STF: Li, Linfeng
Messung:          API-MS (HRMS)
Ionisierung:      ESIpos
Lösungsmittel:    CH2Cl2+CH3CN+CH3COOH
Spektrometer:     Q Exactive Plus Orbitrap
Auswerter:        Kohler (2243)
```

```
Mass to be matched (m/z): 158.154050  Charge: 1

Mass Tolerance: ±0.005000
Restriction of atom numbers:
C       H       N       O
1-100   1-100   1-3     max 10
Number of calculated Formulas:   1

Formula            Diff.(ppm)    theor. m/z
C9 H20 N1 O1            -0.70    158.153939

                    Suggestion:
                    C9H19N1O1   MW 157

                    Characteristicial ions:
                    158 = [157 + H]+
```

```
Mass to be matched (m/z): 172.169580  Charge: 1

Mass Tolerance: ±0.005000
Restriction of atom numbers:
C       H       N       O
1-100   1-100   1-3     max 10
Number of calculated Formulas:   1

Formula            Diff.(ppm)    theor. m/z
C10 H22 N1 O1            0.05    172.169589

                      Suggestion:
                      C10H21N1O1   MW 171

                      Characteristicial ions:
                      172 = [171 + H]+
```

```
Mass to be matched (m/z): 186.185240  Charge: 1

Mass Tolerance: ±0.005000
Restriction of atom numbers:
C       H       N       O
1-100   1-100   1-3     max 10
Number of calculated Formulas:   1

Formula            Diff.(ppm)    theor. m/z
C11 H24 N1 O1           -0.01    186.185239

                    Suggestion:
                    C11H23N1O1 MW 185

                    Characteristicial ions:
                    186 = [185 + H]+
```

```
Mass to be matched (m/z): 200.200900  Charge: 1

Mass Tolerance: ±0.005000
Restriction of atom numbers:
C       H       N       O
1-100   1-100   1-3     max 10
Number of calculated Formulas:   1

Formula            Diff.(ppm)    theor. m/z
C12 H26 N1 O1           -0.06    200.200889

                    Suggestion:
                    C12H25N1O1 MW 199

                    Characteristicial ions:
                    200 = [199 + H]+
```

Entry 2

12.01.2022
File: 156448e-00
Analyse: LIQ-LA-064-01
STF: Li, Linfeng

Messung: API-MS (HRMS)
Ionisierung: ESIpos
Lösungsmittel: CH2Cl2+CH3CN+CH3COOH
Spektrometer: Q Exactive Plus Orbitrap
ELNA:

Auswerter: Kohler (2243)

Mass to be matched (m/z): 214.216490 Charge: 1

Mass Tolerance: ±0.005000
Restriction of atom numbers:

C	H	N	O
1-110	1-100	1-3	1-5

Number of calculated Formulas: 2

Formula	Diff.(ppm)	theor. m/z
C13 H28 N1 O1	0.23	214.216539
C8 H28 N3 O3	-18.55	214.212516

Suggestion:
C13H27N1O1 MW 213

Characteristicial ions:
214 = [213 + H]+

Mass to be matched (m/z): 228.232160 Charge: 1

Mass Tolerance: ±0.005000
Restriction of atom numbers:

C	H	N	O
1-110	1-100	1-3	1-5

Number of calculated Formulas: 2

Formula	Diff.(ppm)	theor. m/z
C14 H30 N1 O1	0.13	228.232189
C9 H30 N3 O3	-17.50	228.228166

Suggestion:
C14H29N1O1 MW 227

Characteristicial ions:
228 = [227 + H]+

Mass to be matched (m/z): 242.247750 Charge: 1

Mass Tolerance: ±0.005000
Restriction of atom numbers:

C	H	N	O
1-110	1-100	1-3	1-5

Number of calculated Formulas: 2

Formula	Diff.(ppm)	theor. m/z
C15 H32 N1 O1	0.37	242.247839
C10 H32 N3 O3	-16.24	242.243816

Suggestion:
C15H31N1O1 MW 241

Characteristicial ions:
242 = [241 + H]+

Entry 3

electrospray-ionization (Sol.: CH2Cl2+CH3CN+CH3COOH)
pos. ions

characteristical ions
142 = [C9H20N1]+

Datum	15.12.2021
File:	156958b-00.RAW
ELNA:	34753
Analyse:	LIQ-LA-064-03
STF: Li, Linfeng	
Ionisierung:	ESIpos
Lösungsmittel:	CH2Cl2+CH3CN+CH3COOH
Spektrometer:	Exactive
Auswerter:	Kampen (2242)

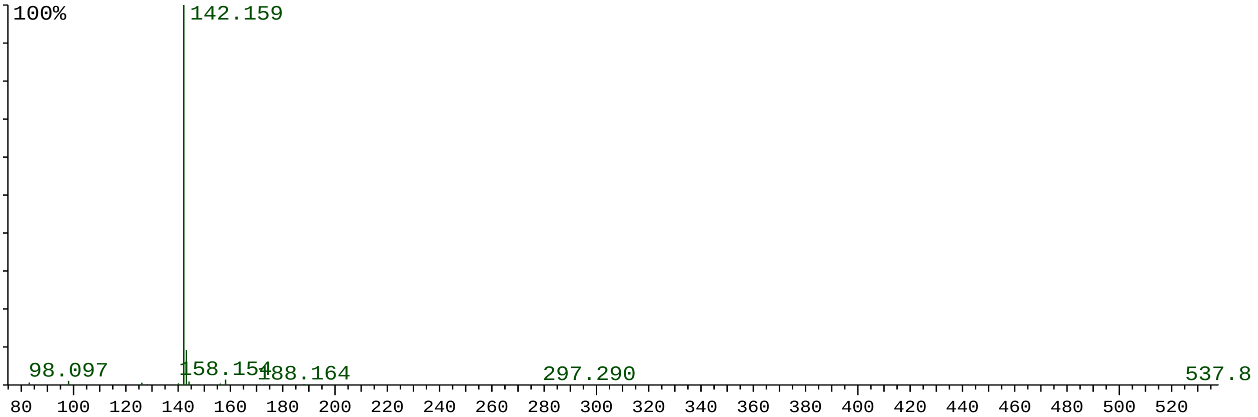

R3: 77(2:56) m(69-77) 3566 86% 156958b-00 * LIQ-LA-064-03/ESIpos/CH2Cl2+CH3CN+CH3COOH/Exact

reduced mass range m/z 150-250

characteristical ions
156 = [C9H18N1O1]+
158 = [C9H20N1O1]+ = [157 + H]+
172 = [C10H21N1O1]+ = [171 + H]+
186 = [C11H24N1O1]+ = [185 + H]+ (low intensity)
200 = [C12H26N1O1]+ = [199 + H]+
214 = [C13H28N1O1]+
228 = [C14H30N1O1]+
242 = [C15H32N1O1]+

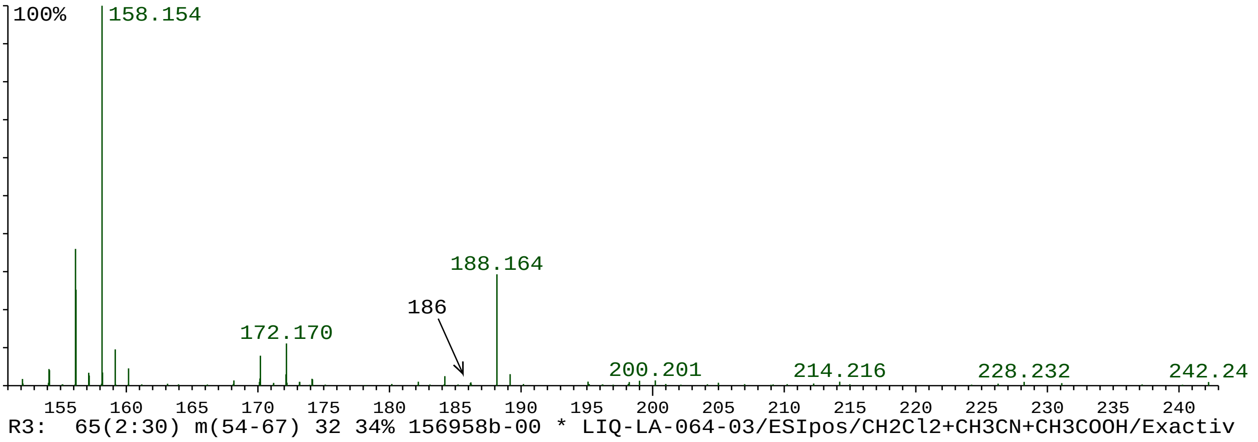

R3: 65(2:30) m(54-67) 32 34% 156958b-00 * LIQ-LA-064-03/ESIpos/CH2Cl2+CH3CN+CH3COOH/Exactiv

Entry 3

15.12.2021
File: 156958b-00.RAW
Analyse: LIQ-LA-064-03
STF: Li, Linfeng

Messung: API-MS (HRMS)
Ionisierung: ESIpos
Lösungsmittel: CH2Cl2+CH3CN+CH3COOH
Spektrometer: Q Exactive Plus Orbitrap

Auswerter: Kohler (2243)

Mass to be matched (m/z): 158.153950 Charge: 1

Mass Tolerance: ±0.005000
Restriction of atom numbers:

C	H	N	O
1-100	1-100	1-3	max 10

Number of calculated Formulas: 1

Formula	Diff.(ppm)	theor. m/z
C9 H20 N1 O1	-0.07	158.153939

Suggestion:
C9H19N1O1 MW 157

Characteristicial ions:
158 = [157 + H]+

Mass to be matched (m/z): 172.169540 Charge: 1

Mass Tolerance: ±0.005000
Restriction of atom numbers:

C	H	N	O
1-100	1-100	1-3	max 10

Number of calculated Formulas: 1

Formula	Diff.(ppm)	theor. m/z
C10 H22 N1 O1	0.28	172.169589

Suggestion:
C10H21N1O1 MW 171

Characteristicial ions:
172 = [171 + H]+

Mass to be matched (m/z): 186.185220 Charge: 1

Mass Tolerance: ±0.005000
Restriction of atom numbers:

C	H	N	O
1-100	1-100	1-3	max 10

Number of calculated Formulas: 1

Formula	Diff.(ppm)	theor. m/z
C11 H24 N1 O1	0.10	186.185239

Suggestion:
C11H23N1O1 MW 185

Characteristicial ions:
186 = [185 + H]+

Mass to be matched (m/z): 200.200840 Charge: 1

Mass Tolerance: ±0.005000
Restriction of atom numbers:

C	H	N	O
1-100	1-100	1-3	max 10

Number of calculated Formulas: 1

Formula	Diff.(ppm)	theor. m/z
C12 H26 N1 O1	0.24	200.200889

Suggestion:
C122H25N1O1 MW 199

Characteristicial ions:
200 = [199 + H]+

Entry 3

12.01.2022
File: 156958c-00
Analyse: LIQ-LA-064-03
STF: Li, Linfeng

Messung: API-MS (HRMS)
Ionisierung: ESIpos
Lösungsmittel: CH2Cl2+CH3CN+CH3COOH
Spektrometer: Q Exactive Plus Orbitrap
ELNA:

Auswerter: Kohler (2243)

Mass to be matched (m/z): 214.216420 Charge: 1

Mass Tolerance: ±0.005000
Restriction of atom numbers:

C	H	N	O
1-110	1-100	1-3	1-5

Number of calculated Formulas: 2

Formula	Diff.(ppm)	theor. m/z
C13 H28 N1 O1	0.55	214.216539
C8 H28 N3 O3	-18.22	214.212516

Suggestion:
C13H27N1O1 MW 213

Characteristicial ions:
214 = [213 + H]+

Mass to be matched (m/z): 228.232130 Charge: 1

Mass Tolerance: ±0.005000
Restriction of atom numbers:

C	H	N	O
1-110	1-100	1-3	1-5

Number of calculated Formulas: 2

Formula	Diff.(ppm)	theor. m/z
C14 H30 N1 O1	0.26	228.232189
C9 H30 N3 O3	-17.37	228.228166

Suggestion:
C14H29N1O1 MW 227

Characteristicial ions:
228 = [227 + H]+

Mass to be matched (m/z): 242.247650 Charge: 1

Mass Tolerance: ±0.005000
Restriction of atom numbers:

C	H	N	O
1-110	1-100	1-3	1-5

Number of calculated Formulas: 2

Formula	Diff.(ppm)	theor. m/z
C15 H32 N1 O1	0.78	242.247839
C10 H32 N3 O3	-15.83	242.243816

Suggestion:
C15H31N1O1 MW 241

Characteristicial ions:
242 = [241 + H]+

Entry 4

Elektrospray-ionisation pos. ions

Characteristicial ions:
142 = [C9H20N1]+

Additionally characteristical ions:
297

12.01.2022
File: E34915a-00.RAW
Analyse: LIQ-LA-064-04
STF: Li, Linfeng

Messung:	API-MS
Ionisierung:	ESIpos
Lösungsmittel:	CH2Cl2+CH3CN+CH3COOH
Spektrometer:	Q Exactive Plus Orbitrap
ELNA:	
Auswerter:	Kohler (2243)

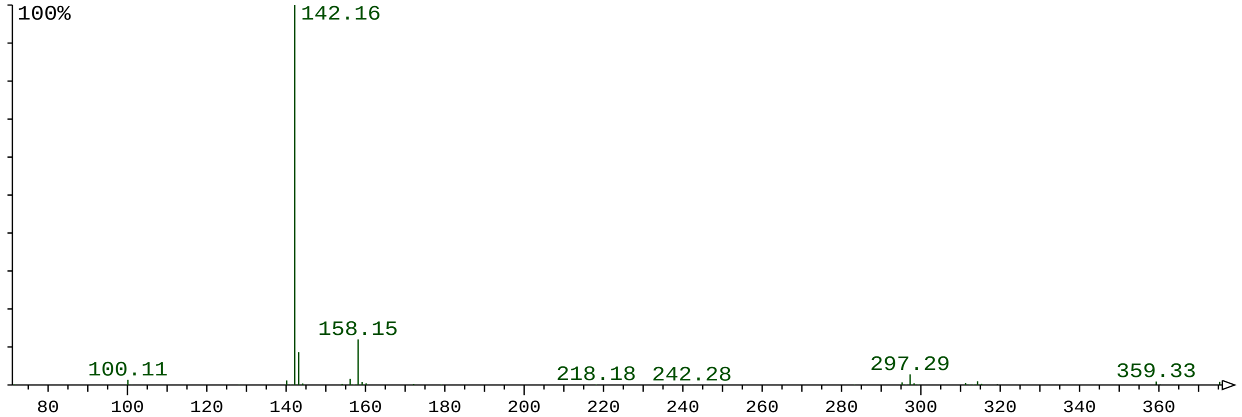

R3: 14(0:30) m(10-15) 68813 72% E34915a-00 * LIQ-LA-064-04/ESIpos/CH2Cl2+CH3CN+CH3OH/Exacti

reduced mass range m/z 145 - 290

Characteristicial ions:
156 = [C9H18N1O1]+
158 = [C9H20N1O1]+ = [157 + H]+

Additionally characteristical ions:
172.13, 240, 269, 283

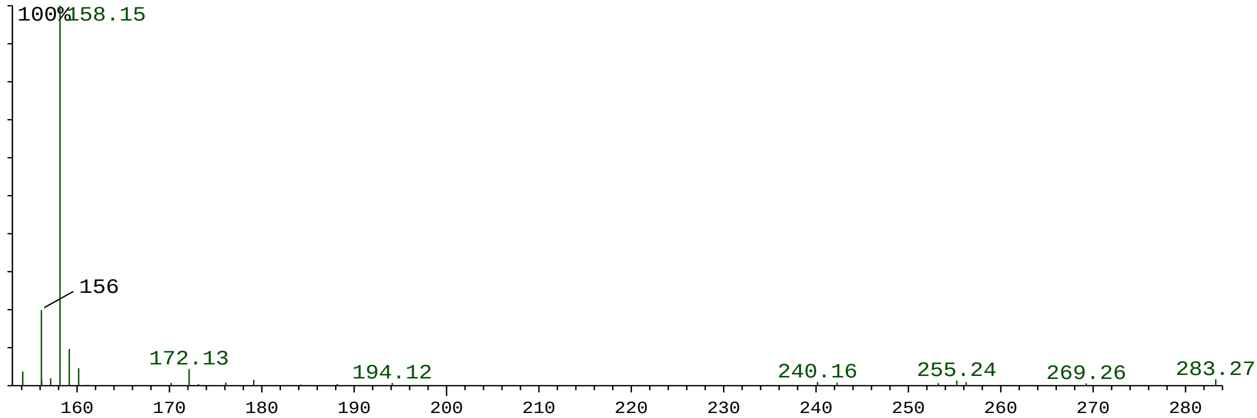

R3: 72(2:34) m(51-76) 6093 63% E34915a-00 * LIQ-LA-064-04/ESIpos/CH2Cl2+CH3CN+CH3OH/Exactiv

Entry 4

Mass to be matched (m/z): 158.153970 Charge: 1

Mass Tolerance: ±0.005000
Restriction of atom numbers:

C	H	N	O
1-110	1-100	1-3	1-5

Number of calculated Formulas: 2

Formula	Diff.(ppm)	theor. m/z
C9 H20 N1 O1	-0.20	158.153939
C4 H20 N3 O3	-25.63	158.149916

Suggestion:
C9H19N1O1 MW 157

Characteristicial ions:
158 = [157 + H]+

Entry 5

electrospray-ionization (Sol.: CH2Cl2+CH3CN+CH3COOH)
pos. ions

characteristical ions
142 = [C9H20N1]+

Datum	7.01.2022
File:	E34883a-00.RAW
ELNA:	
Analyse:	LIQ-LA-069-01
STF: Li, Linfeng	
Ionisierung:	ESIpos
Lösungsmittel:	CH2Cl2+CH3CN+CH3COOH
Spektrometer:	Exactive
Auswerter:	Kampen (2242)

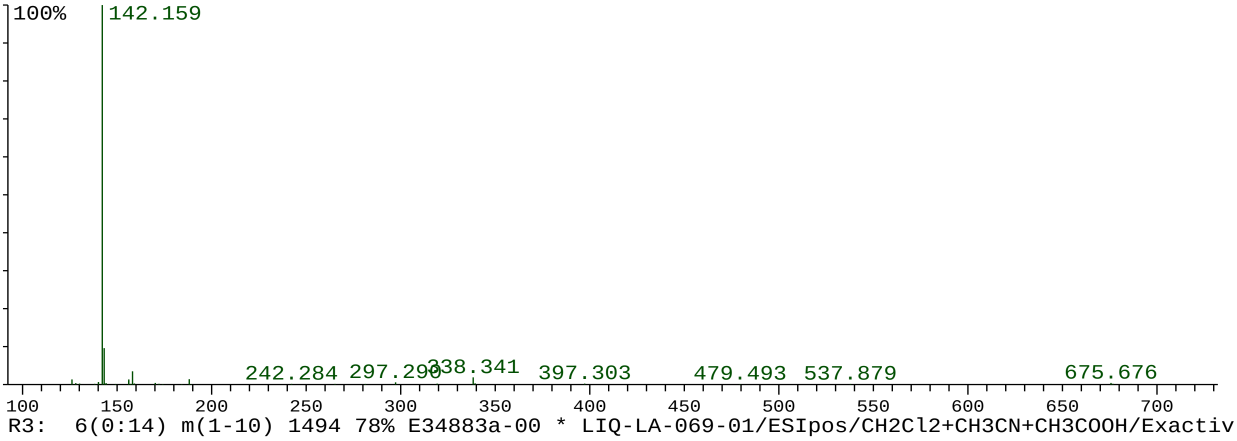

R3: 6(0:14) m(1-10) 1494 78% E34883a-00 * LIQ-LA-069-01/ESIpos/CH2Cl2+CH3CN+CH3COOH/Exactiv

reduced mass range m/z 150-300

characteristical ions
156 = [C9H18N1O1]+ (156.138)
158 = [C9H20N1O1]+ = [157 + H]+

characteristical ions (low intensity)
172 = [C10H22N1O1]+ = [171 + H]+
186 = [C11H24N1O1]+ = [185 + H]+
200 = [C12H26N1O1]+ = [199 + H]+
214 = [C13H28N1O1]+
228 = [C14H30N1O1]+
242 = [C15H32N1O1]+ (242.247)

additional characteristical ions
156 (156.175), 170, 188, 242(242.284), 265, 297

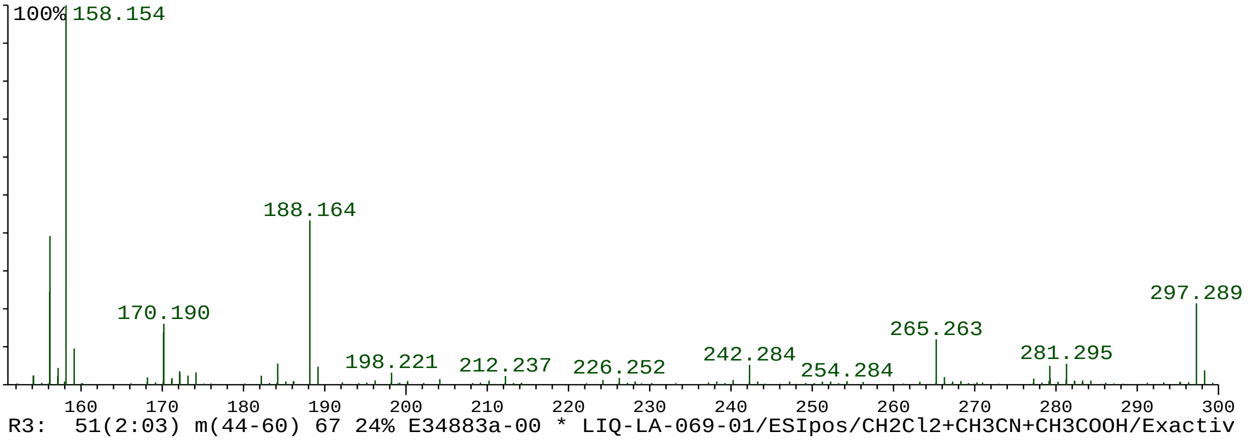

R3: 51(2:03) m(44-60) 67 24% E34883a-00 * LIQ-LA-069-01/ESIpos/CH2Cl2+CH3CN+CH3COOH/Exactiv

Entry 5

Mass to be matched (m/z): 158.153750 Charge: 1

Mass Tolerance: ±0.005000
Restriction of atom numbers:

C	H	N	O
1-100	1-100	1-3	max 10

Number of calculated Formulas: 1

Formula	Diff.(ppm)	theor. m/z
C9 H20 N1 O1	1.19	158.153939

Suggestion:
C9H19N1O1 MW 157

Characteristicial ions:
158 = [157 + H]+

Mass to be matched (m/z): 172.169270 Charge: 1

Mass Tolerance: ±0.005000
Restriction of atom numbers:

C	H	N	O
1-100	1-100	1-3	max 10

Number of calculated Formulas: 1

Formula	Diff.(ppm)	theor. m/z
C10 H22 N1 O1	1.85	172.169589

Suggestion:
C10H21N1O1 MW 171

Characteristicial ions:
172 = [171 + H]+

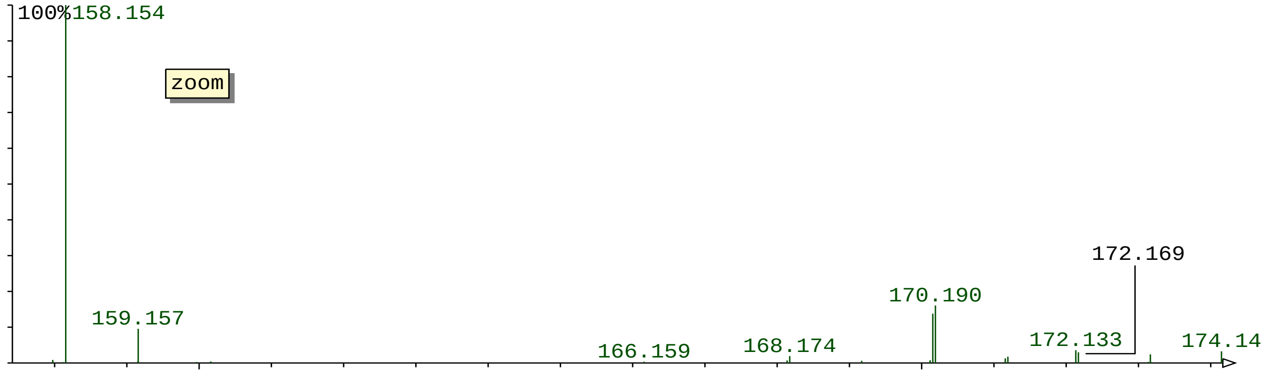

R3: 51(2:03) m(44-60) 67 24% E34883a-00 * LIQ-LA-069-01/ESIpos/CH2Cl2+CH3CN+CH3COOH/Exactiv

Mass to be matched (m/z): 186.184990 Charge: 1

Mass Tolerance: ±0.005000
Restriction of atom numbers:

C	H	N	O
1-100	1-100	1-3	max 10

Number of calculated Formulas: 1

Formula	Diff.(ppm)	theor. m/z
C11 H24 N1 O1	1.34	186.185239

Suggestion:
C11H23N1O1 MW 185

Characteristicial ions:
186 = [185 + H]+

Mass to be matched (m/z): 200.200580 Charge: 1

Mass Tolerance: ±0.005000
Restriction of atom numbers:

C	H	N	O
1-100	1-100	1-3	max 10

Number of calculated Formulas: 1

Formula	Diff.(ppm)	theor. m/z
C12 H26 N1 O1	1.54	200.200889

Suggestion:
C12H25N1O1 MW 199

Characteristicial ions:
200 = [199 + H]+

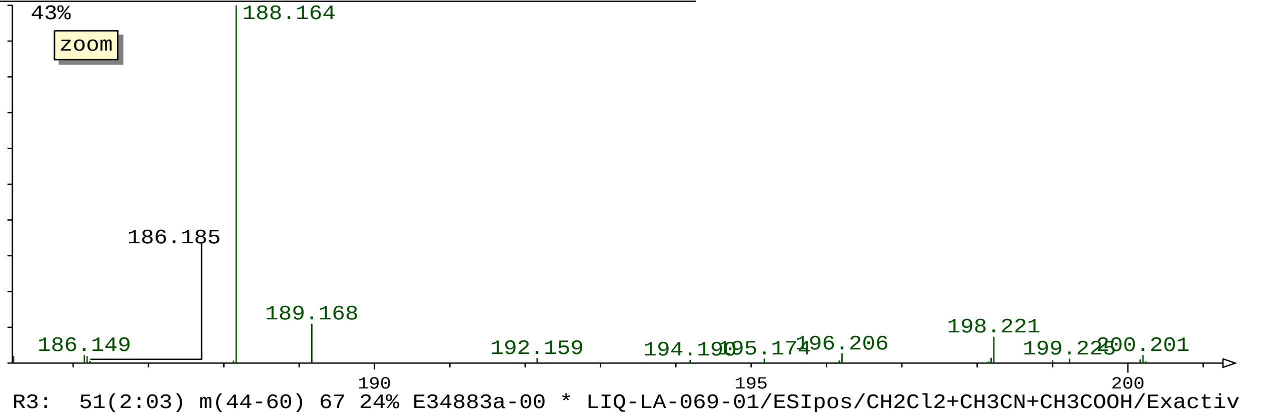

R3: 51(2:03) m(44-60) 67 24% E34883a-00 * LIQ-LA-069-01/ESIpos/CH2Cl2+CH3CN+CH3COOH/Exactiv

Entry 5

Mass to be matched (m/z): 214.216160 Charge: 1

Mass Tolerance: ±0.005000
Restriction of atom numbers:
C H N O
1-110 1-100 1-3 1-5
Number of calculated Formulas: 2

Formula	Diff.(ppm)	theor. m/z
C13 H28 N1 O1	1.77	214.216539
C8 H28 N3 O3	-17.01	214.212516

12.01.2022
File: E34883b-00
Analyse: LIQ-LA-069-01
STF: Li, Linfeng

Messung: API-MS (HRMS)
Ionisierung: ESIpos
Lösungsmittel: CH2CL2+CH3CN+CH3COOH
Spektrometer: Q Exactive Plus Orbitrap
ELNA:

Auswerter: Kohler (2243)

Suggestion:
C13H27N1O1 MW 213

Characteristicial ions:
214 = [213 + H]+

Mass to be matched (m/z): 228.231820 Charge: 1

Mass Tolerance: ±0.005000
Restriction of atom numbers:
C H N O
1-110 1-100 1-3 1-5
Number of calculated Formulas: 2

Formula	Diff.(ppm)	theor. m/z
C14 H30 N1 O1	1.62	228.232189
C9 H30 N3 O3	-16.01	228.228166

Suggestion:
C14H29N1O1 MW 227

Characteristicial ions:
228 = [227 + H]+

Mass to be matched (m/z): 242.247360 Charge: 1

Mass Tolerance: ±0.005000
Restriction of atom numbers:
C H N O
1-110 1-100 1-3 1-5
Number of calculated Formulas: 2

Formula	Diff.(ppm)	theor. m/z
C15 H32 N1 O1	1.98	242.247839
C10 H32 N3 O3	-14.63	242.243816

Suggestion:
C15H31N1o1 MW 241

Characteristicial ions:
242 = [241 + H]+

Entry 6

Elektrospray-ionisation pos. ionschi

Characteristicial ions:
142 = [C9H20N1]+

Additionally characteristical ions:
144.98 (Cu1),

2.08.2022
File: E38432a-00.RAW
Analyse: LIQ-LA-084-01
STF: Li, Linfeng

Messung: API-MS
Ionisierung: ESIpos
Lösungsmittel: CH2Cl2+CH3CN+CH3COOH
Spektrometer: Q Exactive Plus Orbitrap

Auswerter: Kohler (2243)

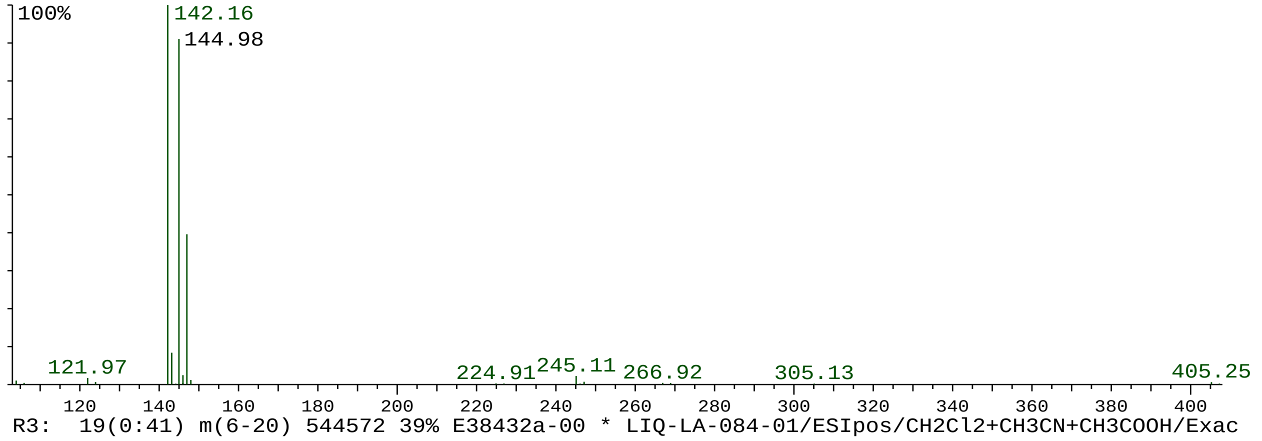

R3: 19(0:41) m(6-20) 544572 39% E38432a-00 * LIQ-LA-084-01/ESIpos/CH2Cl2+CH3CN+CH3COOH/Exac

reduced mass range m/z 150 - 250

Characteristicial ions:
156 = [C9H18N1O1]+
158 = [C9H20N1O1]+ = [157 + H]+

Characteristicial ions (very low intensity):
172 = [C10H22N1O1]+ = [171 + H]+ ; overlapping with other ions

Additionally characteristical ions:
163 (Cu1), 204 (Cu1), 225 (Cu2), 245 (Cu1)

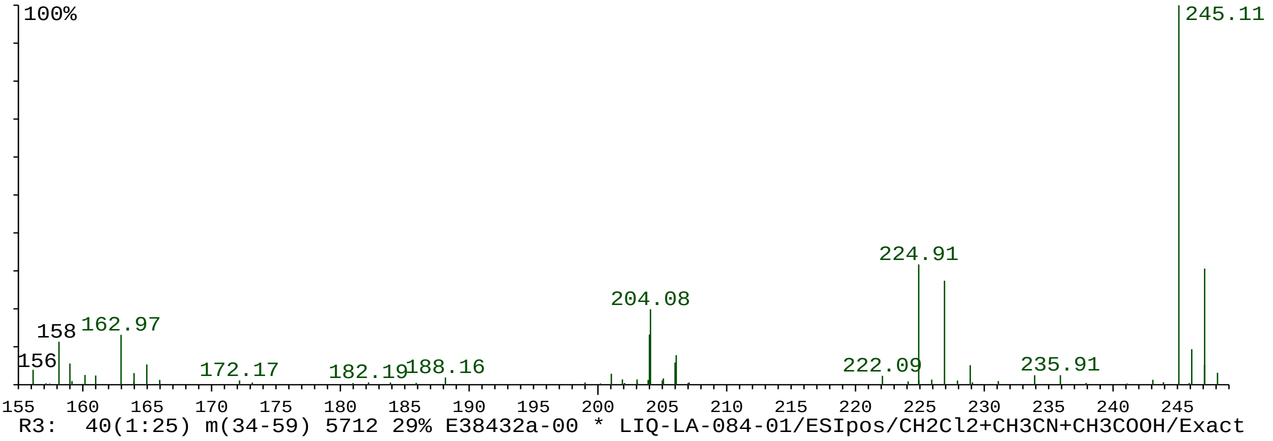

R3: 40(1:25) m(34-59) 5712 29% E38432a-00 * LIQ-LA-084-01/ESIpos/CH2Cl2+CH3CN+CH3COOH/Exact

Entry 6

Mass to be matched (m/z): 158.153930 Charge: 1

Mass Tolerance: ±0.005000
Restriction of atom numbers:

C	H	N	O
1-130	1-100	1-32	1-8

Number of calculated Formulas: 2

Formula	Diff.(ppm)	theor. m/z
C9 H20 N1 O1	0.05	158.153939
C4 H20 N3 O3	-25.38	158.149916

2.08.2022
File: E38432a-00.RAW
Analyse: LIQ-LA-084-01
STF: Li, Linfeng

Messung:	API-MS (HRMS)
Ionisierung:	ESIpos
Lösungsmittel:	CH2Cl2+CH3CN+CH3COOH
Spektrometer:	Q Exactive Plus Orbitrap
Auswerter:	Kohler (2243)

Suggestion:
C9H19N1O1 MW 157

Characteristicial ions:
158 = [157 + H]+
